# Decisions on the allocation of intensive care resources in the context of the COVID-19 pandemic: Clinical and ethical recommendations of DIVI, DGINA, DGAI, DGIIN, DGNI, DGP, DGP and AEM

**DOI:** 10.1007/s00063-020-00709-9

**Published:** 2020-07-29

**Authors:** Georg Marckmann, Gerald Neitzke, Jan Schildmann, Andrej Michalsen, Jochen Dutzmann, Christiane Hartog, Susanne Jöbges, Kathrin Knochel, Guido Michels, Martin Pin, Reimer Riessen, Annette Rogge, Jochen Taupitz, Uwe Janssens

**Affiliations:** 1 Institute of Ethics, History, and Theory of Medicine, Ludwig-Maximilians-University Munich, Munich, Germany; 2 Institute for History, Ethics and Philosophy of Medicine, Hannover Medical School, Hannover, Germany; 3 Institute for the History and Ethics of Medicine, Interdisciplinary Center for Health Sciences, Martin-Luther-University Halle-Wittenberg, Halle (Saale), Germany; 4 Clinic for Anaesthesiology, Intensive Care, Emergency Care and Analgesic Therapy, Hospital Tettnang, Tettnang, Germany; 5 Medical University and Polyclinic for Internal Medicine III, University Hospital Halle (Saale), Halle (Saale), Germany; 6 Clinic for Anaesthesiology and Intensive Care, Charité Medical School Berlin, Berlin, Germany; 7 Hospital Bavaria Kreischa, Kreischa, Germany; 8 Institute for Biomedical Ethics and History of Medicine, University Zürich, Zürich, Switzerland; 9 Paediatric Clinic and Paediatric Polyclinic Dr. von Haunerschen Kinderspital, University Hospital Munich, Munich, Germany; 10 Department for Acute and Emergency Medicine, St. Antonius Hospital Eschweiler, Eschweiler, Germany; 11 Central Interdisciplinary Emergency Department, Florence-Nightingale-Hospital Kaiserswerther Diakonie, Düsseldorf, Germany; 12 Medical Intensive Care Unit 93, Department for Internal Medicine, University Hospital Tübingen, Tübingen, Germany; 13 Division Ethics of Medicine, Christian-Albrechts-University Kiel, Kiel, Germany; 14 Legal Research Department, University Mannheim, Mannheim, Germany; 15 Medical Clinic and Medical Intensive Care Medicine, St. Antonius Hospital Eschweiler, Dechant-Deckers-Str. 8, 52249 Eschweiler, Germany

**Keywords:** Prioritisation, Triage, Scarcity, Justice, Intensive care medicine, Priorisierung, Triage, Ressourcenmangel, Gerechtigkeit, Intensivmedizin

## Abstract

In view of the globally evolving coronavirus disease (COVID-19) pandemic, German hospitals rapidly expanded their intensive care capacities. However, it is possible that even with an optimal use of the increased resources, these will not suffice for all patients in need. Therefore, recommendations for the allocation of intensive care resources in the context of the COVID-19 pandemic have been developed by a multidisciplinary group of authors with the support of eight scientific medical societies. The recommendations for procedures and criteria for prioritisations in case of resource scarcity are based on scientific evidence, ethicolegal considerations and practical experience. Medical decisions must always be based on the need and the treatment preferences of the individual patient. In addition to this patient-centred approach, prioritisations in case of resource scarcity require a supraindividual perspective. In such situations, prioritisations should be based on the criterion of clinical prospect of success in order to minimize the number of preventable deaths due to resource scarcity and to avoid discrimination based on age, disabilities or social factors. The assessment of the clinical prospect of success should take into account the severity of the current illness, severe comorbidities and the patient’s general health status prior to the current illness.

**Electronic supplementary material:**

The online version of this article (10.1007/s00063-020-00709-9) includes Fig. 1 “Documentation support for prioritisation in case of resource scarcity” and Fig. 2 “Flowchart—decision-making in the case of insufficient intensive care resources” for download. Contributions and additional material are available at www.springermedizin.de. Please enter the article title in the search field, the additional material can be found under “Ergänzende Inhalte”.

## Abstract (de)

Angesichts der beginnenden COVID-19-Pandemie haben die deutschen Krankenhäuser ihre intensivmedizinischen Kapazitäten erhöht. Trotz optimaler Nutzung erscheint es möglich, dass die intensivmedizinischen Ressourcen nicht mehr für alle Patienten ausreichen, die ihrer bedürfen. Vor diesem Hintergrund wurden von einer multidisziplinären Autorengruppe und mit Unterstützung von acht medizinisch-wissenschaftlichen Fachgesellschaften Empfehlungen zur Verteilung intensivmedizinischer Ressourcen im Kontext der COVID-19-Pandemie erarbeitet. Die Empfehlungen zu Verfahren und Kriterien für Priorisierungsentscheidungen bei Ressourcenknappheit wurden auf Grundlage der verfügbaren wissenschaftlichen Evidenz, ethischer und rechtlicher Überlegungen sowie praktischen Erfahrungen erstellt. Medizinische Entscheidungen müssen sich immer am Bedarf und den Behandlungspräferenzen des einzelnen Patienten orientieren. Ergänzend zu dieser patientenzentrierten Betrachtung kommt mit der Priorisierung bei absoluter Mittelknappheit eine überindividuelle Perspektive hinzu. In dieser Situation sollte die Priorisierung anhand des Kriteriums der Erfolgsaussicht einer Behandlung erfolgen, um die Anzahl der vermeidbaren Todesfälle zu minimieren und eine Diskriminierung von Patienten aufgrund des Alters, von Behinderungen oder des sozialen Status zu vermeiden. Die Einschätzung der klinischen Erfolgsaussicht berücksichtigt den Schweregrad der aktuellen Erkrankung, das Vorliegen schwerer Komorbiditäten und den allgemeinen prämorbiden Gesundheitsstatus des Patienten.

## Societies involved

- German Interdisciplinary Association for Intensive Care and Emergency Medicine (DIVI)
- German Society for Interdisciplinary Emergency and Acute Medicine (DGINA)
- German Society for Anaesthesiology and Intensive Care Medicine (DGAI)
- German Society for Internal Intensive Care Medicine and Emergency Medicine (DGIIN)
- German Society for Neurointensive and Emergency Medicine (DGNI)
- German Society for Pulmonology and Respiratory Medicine (DGP)
- German Society for Palliative Medicine (DGP)
- German Academy of Ethics in Medicine (AEM)

## 1. Background

In the face of the globally evolving Coronavirus Disease 2019 (COVID‑19) pandemic, German hospitals rapidly expanded their intensive care capacities. However, it seems still possible that even an optimal use of the increased intensive care resources will not be sufficient to treat all patients who require them [13, 19]. Consequently, the need for guidance on the potentially resulting dilemmas prompted the authors, in coordination with the boards of directors of the participating scientific societies, to develop recommendations for the allocation of intensive care resources in the context of the COVID-19 pandemic [17, 18]. The guidance is intended to support responsible decision makers with medically and ethically justified criteria and procedures. Experts from clinical emergency medicine, intensive care medicine, medical ethics, law, and further disciplines were involved in drafting the recommendations. Several experts reviewed a prior version; they are listed at the end of the document.

These recommendations will be further developed on the basis of new scientific evidence, practical experience and other relevant developments. The current German version can be found at www.divi.de and www.awmf.org (S1 guideline, register number 040-013) [8]. Comments on the recommendations are explicitly encouraged.

## 2. General principles of decision-making

Medical decisions must always be based on the needs of the individual patient (see 2.1). In addition to this patient-centred approach, prioritisation in the event of a resource shortage requires a supra-individual perspective (see 2.2).

### 2.1 The basis for individual, patient-centred decisions

The clinical indications and the patient’s autonomous choices form the basis for every patient-centred decision [12, 15, 16]:

- Intensive care treatment is not indicated, if
  - The dying process has started inexorably
  - The treatment is considered medically inappropriate because no medical improvement or stabilisation is expected or
  - Survival can only be achieved by permanent intensive care treatment.
- Patients who refuse intensive care are not treated in the intensive care unit. The refusal can be based on currently expressed wishes, previously documented wishes (e.g. in an advance directive), afore orally expressed wishes or substituted judgement. The patient’s autonomous choice can be executed by the patient herself or by her legal representative.

### 2.2 Additional basis for decisions under conditions of resource scarcity

If the available resources are *not* sufficient—neither in-house nor regionally or transregional—inevitably a decision has to be made which critically ill patients should be treated with intensive care and which should not (or no longer) be treated with intensive care. If resources are scarce, the following situations may develop:

- No intensive care resources available, but resources in the emergency room (e.g. temporary ventilation therapy until transfer)
- No intensive care resources available, no resources in the emergency room, but resources in surrounding hospitals (e.g. coordination by a regional task force of the respective crisis management team)
- No intensive care resources available, no resources in the emergency room, no accessible additional resources.

If no resources are available after checking the above-mentioned provisions, a deviation from the usual patient-centred approach to treatment decisions becomes necessary. This poses enormous emotional and moral challenges for the treatment team.

In this case, decisions about the allocation of the scarce resources must be made in analogy to triage decisions in disaster medicine. This prioritisation requires transparent, medically and ethically well-founded criteria [11, 14, 21, 22]. Such an approach can support the hospital staff involved and increase public trust in the hospitals’ crisis management. The prioritisation is explicitly *not* intended to assess the value of people or human lives. Instead, the prioritisation shall allow as many patients as possible to benefit from the (limited) medical resources under the conditions of a pandemic crisis.

The prioritisation of patients should therefore be based on the **criterion of clinical prospect of success **[18]. Accordingly, those patients who have a very low chance of survival will—if unavoidable—*not* be treated with intensive care. Priority will be given to those patients who are more likely to survive when receiving intensive care. The clinical prospect of success must be assessed as carefully as possible for each individual patient.

Prioritisations should always

- Consider all patients who need intensive care, regardless of where they are being cared for (general ward, emergency room/intermediate care, intensive care unit).

According to the principle of equality, prioritisations

- Are not justifiable exclusively within the group of COVID-19 patients
- Are not permitted on the basis of calendar age, social characteristics, or specific underlying illnesses or disabilities.

Note: According to German constitutional law, human lives must not be weighed against other human lives. At the same time, treatment resources must be utilized responsibly. These recommendations are based on what the authors consider to be the most justifiable ethical principles in the situation of tragic choices. They shall minimise the number of preventable deaths due to resource scarcity. A final legal assessment is beyond the scope of these recommendations.

## 3. Procedures and criteria for prioritisation in case of resource scarcity

The following procedures for prioritisations only apply if intensive care capacities are *not* sufficient for all patients.

In clinical practice, a distinction can be made between:

1. Decisions about for which patients intensive care treatment should be initiated and
2. Decisions about for which patients ongoing intensive care treatment should be withdrawn.

Both decisions are related, and the following criteria and procedures apply to both.

The decisions have to be re-evaluated regularly, at intervals appropriate for COVID-19, and adjusted, where applicable; in particular:

1. In the case of clinically relevant changes in the patient’s condition and/or
2. When the ratio of needed to available resources has changed.

It must be ensured that appropriate treatment is available for those patients who cannot, or can no longer, be treated in intensive care units [6].

### 3.1 Decision-making process

A predefined decision-making process with clearly assigned responsibilities is a prerequisite for consistent, fair, medically and ethically well-founded prioritisation. Therefore, whenever possible, the decisions should be made according to the **multiple-eyes principle** including:

- Two physicians experienced in intensive care medicine, if possible, including practitioners from the involved clinical departments and specialties,
- One experienced member of the nursing staff, if possible, and
- If necessary, representatives from other disciplines (e.g. clinical ethics).

**Fig. 1:**
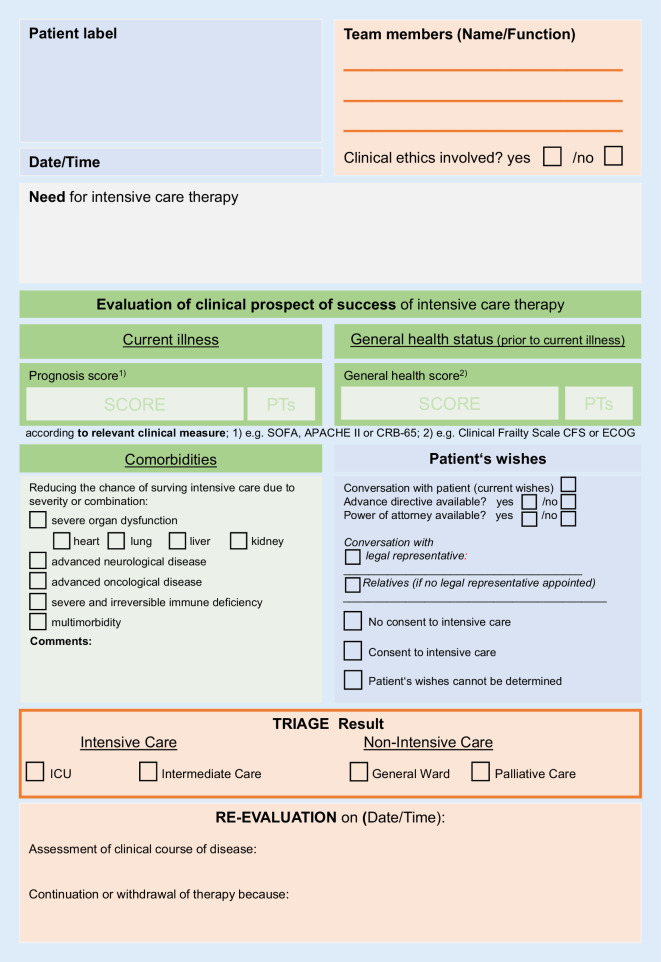
Documentation support for prioritisation in case of resource scarcity. *SOFA* Sepsis-related Organ Failure Assessment, *APACHE II* Acute Physiology and Chronic Health Disease Classification System II, *CRB-65* Confusion, Respiratory rate, Blood pressure, *ECOG* Eastern Cooperative Oncology Group

Staff from both the emergency room and the intensive care unit should be involved. Preferably, decisions should be made by consensus. Hospitals should establish appropriate procedures for dealing with disagreement. Decisions should be made within the multiprofessional and interdisciplinary teams, communicated transparently to patients, relatives (as far as possible) and legal representative(s). Decisions should be documented appropriately (Fig. 1).

### 3.2 Criteria for prioritisations

Prioritisations must be made based on the best available information. This includes:

1. Information on the patient’s current clinical condition
2. Information about the patient’s wishes (current/declared in advance/previously orally expressed or presumed)
3. Medical history/clinical assessment of comorbidities
4. Medical history and clinical assessment of the general health status (including frailty, e.g. according to the Clinical Frailty Scale)
5. Laboratory parameters regarding points 1 and 3, if available
6. Scores with prognostic relevance (e.g. Sepsis-related organ failure assessment [SOFA] score) [20]

In addition, current experiences and knowledge must be taken into account, in particular regarding treatment options and chances of success in COVID-19.

**Fig. 2:**
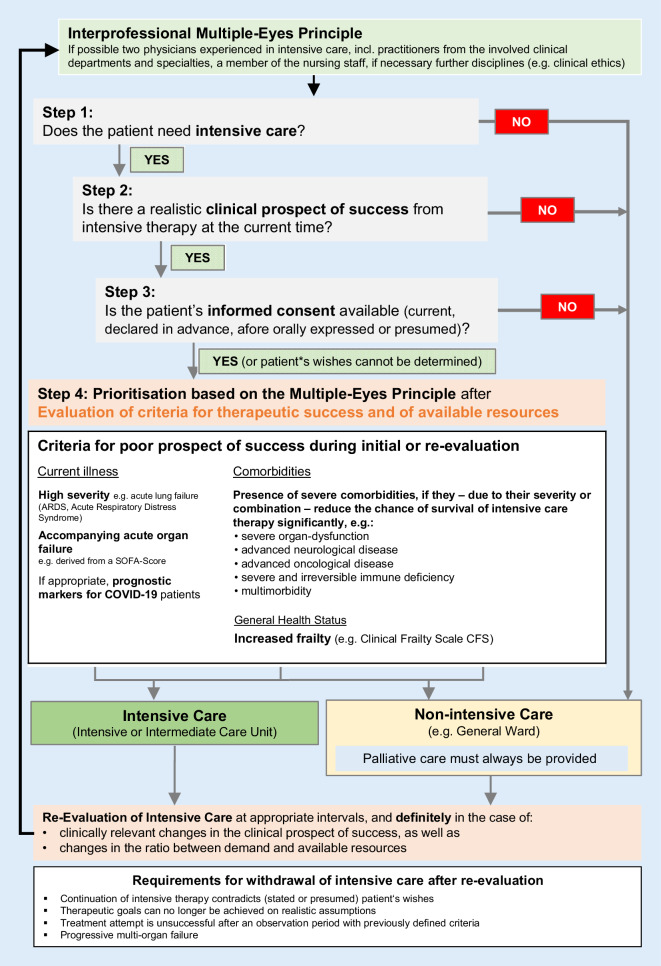
Flowchart—decision-making in the case of insufficient intensive care resources

The decision-making steps and the criteria to be used are as follows (see also flowchart in Fig. 2).

#### 3.2.1 Decisions on admission to the intensive care unit (ICU)

**Step 1: **Assessment of** the need for intensive care treatment**

- Respiratory or haemodynamic failure

*Results:*

- *Intensive care treatment required *→* step 2*
- *Intensive care treatment not required *→* transfer, for instance, to general ward*

**Step 2:** Assessment of the patient’s individual **clinical prospect of success**, i.e. the probability of surviving the current illness through intensive care treatment.

The diseases and conditions mentioned below do not represent* exclusion criteria* for treatment in contrast to other triage protocols [3]. Rather, an overall assessment should consider all important factors influencing the prospect of success (current illness, comorbidities, general health status). Pre-existing diseases are only relevant if they influence the probability of surviving the current illness. This assessment also serves as the basis for any prioritisation which may be necessary (step 4).

The following **criteria**—depending on the degree of their expression—are indicators of a **poor clinical prospect of success** of intensive care treatment:

- *Current illness*
  - Severity of the current main disease or injury (e.g. severe Acute Respiratory Distress Syndrome [ARDS], severe polytrauma, severe brain damage)
  - Concurring acute organ failure (e.g. determined with the SOFA score)
  - Prognostic markers for COVID-19 patients (as soon as they are available and validated)
- *Comorbidities*
  Existing severe comorbidities if their severity—alone or in combination—significantly reduces the probability of surviving the intensive care treatment:
  - Severe organ dysfunction with a limited life expectancy, e.g. advanced heart failure; advanced lung diseases, e.g. advanced Chronic Obstructive Pulmonary Disease (COPD) or chronic respiratory insufficiency requiring ventilation; advanced kidney failure; advanced liver failure
  - Advanced neurological diseases
  - Advanced cancer
  - Severe and irreversible immune deficiency
  - Multimorbidity
- *General health status (prior to current illness)*
  - Frailty (e.g. according to the Clinical Frailty Scale as part of a geriatric assessment)

*Results:*

- *Intensive care treatment medically inappropriate (lack of medical indication) *→* no admission to ICU, adequate alternative treatment including palliative care*
- *Intensive care treatment has sufficient prospect of success *→* step 3*

**Step 3: **Check** informed consent **to intensive care treatment (current, declared in advance, previously orally expressed or presumed patient wishes) after disclosure of medical information and prospect of success to the patient or her legal representative.

*Results:*

- *No informed consent *→* no intensive care treatment, but adequate alternative treatment including palliative care*
- *Informed consent given or patient’s wishes cannot be determined *→* step 4*

**Step 4: Prioritisation **(only in case of resource scarcity)

- After assessing the prospects of success of possible intensive care treatment
- Regarding a realistically achievable patient-centred treatment goal
- Compared to the prospect of success of intensive care treatment for other patients
- Taking into account the available resources

*Results:*

- *High priority for treatment *→* admission to ICU*
- *Low priority for treatment *→* no admission to ICU, adequate alternative treatment including palliative care*

#### 3.2.2 Decisions on changing the therapeutic goal during ongoing intensive care treatment (re-evaluation)

For reasons of justice, all patients who require intensive care treatment should be considered equally in the prioritisation. In Germany, this may touch legal limits if intensive care measures are withdrawn in the context of prioritisation (see the ad hoc statement of the German Ethics Council on the Corona crisis) [10]. As there are currently no specific legal regulations in Germany, the decision-makers bear the responsibility for these decisions.

A re-evaluation should be undertaken and documented if there are changes in the patient’s state of health and/or the available resources. Notwithstanding the above, the indication for continuing intensive care treatment must be reviewed carefully on a regular basis.

**Step 1**: **Patient-centred evaluation** of intensive care treatment

**Result 1: **Patient meets criteria for transfer or discharge

- Breathing and circulation are stabilised, transfer or discharge from ICU is possible
  →* Transfer* *patient from ICU*

**Result 2: **Requirements for continuing intensive care are met

- Stabilisation or improvement of organ function(s) are expected or have occurred; further intensive care treatment required
- Therapeutic goal still seems realistic
  →* Continue to step 2: patient included in process of prioritisation*

**Result 3: **Requirements for withdrawal of intensive care are met, e.g.

- Continuing intensive care treatment contradicts the (current, declared in advance, previously orally expressed, presumed) patient’s wishes
- Therapeutic goal is no longer achievable on realistic assumptions
- Treatment attempt is unsuccessful after agreed time interval with previously defined criteria
- Progressive multiple organ failure
  →* Change treatment goal: transfer patient from ICU to receive alternative treatment outside the ICU, including palliative care*

**Step 2**: **Prioritising **of intensive care

- On the basis of the prospect of success of ongoing intensive care treatment, taking into account, amongst other considerations:
  - Organ function during intensive care treatment
  - Trajectory of the underlying disease
  - Response to treatment to-date
- Compared to other patients in need for intensive care treatment
- Taking into account the available resources

*Results:*

- *High priority for treatment *→* continue intensive care*
- *Low priority for treatment *→* termination of intensive care treatment, adequate alternative treatment including palliative care*

### 3.3 Further situations relevant to prioritisation

#### 3.3.1 Preclinical decisions (e.g. nursing homes)

In the preclinical field, the careful assessment of indicators for hospital admission with possible intensive care treatment and the determination of the patient’s preferences are of paramount importance. However, any prioritisation of patients must take place in the relevant in-patient facilities; the emergency physician and paramedic staff only have limited diagnostic options and do not have sufficient information about the current availability of intensive care capacities and allocation criteria [2].

Where possible, decisions about whether hospital admission and, if necessary, transfer to an ICU, is medically indicated and/or wanted by the person concerned in case of a health deterioration, should be determined in advance. This process should involve the general practitioner and be documented reliably [9].

#### 3.3.2 Decisions on the general ward

If COVID-19 patients are initially admitted to a general ward, it should be assessed and documented as early as possible whether intensive care treatment in case of a critically deteriorating health status is (a) medically indicated and/or (b) in accordance with the patient’s preferences. Here, too, the multiple-eyes principle and the support of the treating staff by experienced specialists are required to ease the burden on the intensive care team [15].

## 4. Support options for all staff members

Triage decisions can be a major challenge and burden for the staff involved. Support for the decision-making process and communication of the decision, as well as guidelines for psychosocial support, can be found in the following sources (with link to the respective websites):

Ethical support services: discussion paper of German Academy of Ethics in Medicine (AEM) on the role of ethics committees and other ethics support services in the context of prioritisation [1].

Communication: Hospitals and other relevant institutions should develop strategies for communicating with patients and next of kin in preparation for a medical crisis [4].

Psychosocial support: Regarding psychosocial support for both medical and nursing staff, and patients and next of kin refer to the recommendations by DIVI and DGP [5, 7].

### Infobox Notes on the preparation of these recommendations

*Reviewing experts*

Claudia Bausewein, Julian Bösel, Michael Bucher, Hartmut Bürkle, Hilmar Burchardi, Alena Buyx, Stefan Dinges, Christoph Dodt, Gunnar Duttge, Clemens Eickhoff, Frank Erbguth, Andreas Frewer, Georg Gahn, Steffen Grautoff, Tanja Krones, Stefan Meier, Michael Mohr, Friedemann Nauck, Wiebke Nehls, Benedikt Pannen, Stephan Prückner, Lukas Radbruch, Annette Riedel, Fred Salomon, Oliver Sakowitz, Jürgen in der Schmitten, Anna-Henrikje Seidlein, Alfred Simon, Ralf Stoecker, Herwig Stopfkuchen, Daniel Strech, Jochen Vollmann, Christian Waydhas, Eva Winkler, Bernhard Zwißler.

*Acknowledgements*

The authors would like to thank Prof. Doris Schroeder and Dr. Kate Chatfield (University of Central Lancashire, UK) for providing a translation as basis for this English version of the recommendations.

The authors also thank the numerous commentators for their helpful feedback on the first version of these recommendations. Suggestions have been carefully checked and considered as reflected in the second version.

## Caption Electronic Supplementary Material

Fig. 1 Documentation support for prioritisation in case of resource scarcity

Fig. 2 Flowchart—decision-making in the case of insufficient intensive care resources

## References

[1] Akademie für Ethik in der Medizin (AEM) (2020) Möglichkeiten und Grenzen von Ethikberatung im Rahmen der COVID-19-Pandemie (Stand: 26.03.2020). Ein Diskussionspapier der Akademie für Ethik in der Medizin. https://www.aem-online.de/index.php?id=163. Accessed 30 June 2020 10.1007/s00481-020-00580-4 PMC7189629 32351259

[2] Bundesvereinigung der Arbeitsgemeinschaften Notärzte Deutschlands (BAND) e. V. (2020) Leitplanken für Notärztinnen und Notärzte bei der Zuteilung von Behandlungsressourcen im Kontext der COVID-19-Pandemie. http://www.band-online.de/Leitplanken_fuer_Notaerztinnen_und_Notaerzte_bei_der_Zuteilung_von_Behandlungsressourcen_im_Kontext_der_COVID-19-Pandemie_8465.html. Accessed 30 June 2020

[3] Cheung WK, Myburgh J, Seppelt IM (2012). A multicentre evaluation of two intensive care unit triage protocols for use in an influenza pandemic. Med J Aust.

[4] Deutsche Gesellschaft für Palliativmedizin (2020) Covid-19 kompatible Kommunikation. https://www.dgpalliativmedizin.de/neuigkeiten/covid-19-hinweise-institutionen.html. Accessed 30 June 2020

[5] Deutsche Gesellschaft für Palliativmedizin (2020) Empfehlungen zur Unterstützung von belasteten, schwerstkranken, sterbenden und trauernden Menschen in der Corona Pandemie aus palliativmedizinischer Perspektive. https://www.dgpalliativmedizin.de/neuigkeiten/empfehlungen-der-dgp.html. Accessed 30 June 2020 10.1007/s00482-020-00483-9 PMC7265165 32488422

[6] Deutsche Gesellschaft für Palliativmedizin (2020) Handlungsempfehlungen zur Therapie von Patient*innen mit COVID-19 aus palliativmedizinischer Perspektive 2.0. https://www.dgpalliativmedizin.de/neuigkeiten/empfehlungen-der-dgp.html. Accessed 30 June 2020

[7] Deutsche interdisziplinäre Vereinigung für Intensiv- und Notfallmedizin (DIVI) (2020) Klinische psychosoziale Notfallversorgung im Rahmen von COVID-19 – Handlungsempfehlungen. https://www.divi.de/register/aktuelle-informationen. Accessed 30 June 2020

[8] Deutsche Interdisziplinäre Vereinigung für Intensiv- und Notfallmedizin e. V. (DIVI) (2020) Entscheidungen über die Zuteilung von Ressourcen in der Notfall und der Intensivmedizin im Kontext der COVID-19-Pandemie – Klinisch-ethische Empfehlungen. https://www.awmf.org/leitlinien/detail/ll/040-013.html. Accessed 30 June 2020

[9] Deutsche interprofessionelle Vereinigung Behandlung im Voraus Planen (2020) Ambulante patientenzentrierte Vorausplanung für den Notfall. Ein Leitfaden aus Anlass der Covid-19-Pandemie. https://www.div-bvp.de/. Accessed 30 June 2020

[10] Deutscher Ethikrat (2020) Solidarität und Verantwortung in der Corona-Krise. Ad-hoc-Empfehlung. https://www.ethikrat.org/publikationen/kategorie/ad-hoc-empfehlungen/. Accessed 30 June 2020 10.1007/s00350-020-5563-6 PMC7236439 32454555

[11] Emanuel EJ, Persad G, Upshur R (2020). Fair allocation of scarce medical resources in the time of Covid-19. N Engl J Med.

[12] Janssens U, Burchardi N, Duttge G (2012). Therapiezieländerung und Therapiebegrenzung in der Intensivmedizin. DIVI.

[13] Kain T, Fowler R (2019). Preparing intensive care for the next pandemic influenza. Crit Care.

[14] Nates JL, Nunnally M, Kleinpell R (2016). ICU admission, discharge, and triage guidelines: a framework to enhance clinical operations, development of institutional policies, and further research. Crit Care Med.

[15] Neitzke G, Boll B, Burchardi H (2017). Dokumentation Therapiebegrenzung – Empfehlung der Sektion Ethik der Deutschen Interdisziplinäre Vereinigung für Intensiv und Notfallmedizin (DIVI) unter Mitarbeit der Sektion Ethik der Deutschen Gesellschaft für Internistische Intensivmedizin und Notfallmedizin (DGIIN). Med Klin Intensivmed Notfmed.

[16] Neitzke G, Burchardi H, Duttge G (2016). Grenzen der Sinnhaftigkeit von Intensivmedizin. Med Klin Intensivmed Notfmed.

[17] Österreichische Gesellschaft für Anästhesiologie Reanimation und Intensivmedizin (ÖGARI) (2020) Allokation intensivmedizinischer Ressourcen aus Anlass der Covid-19-Pandemie. https://www.oegari.at/aktuelles.php. Accessed 30 June 2020

[18] Schweizerische Akademie der Medizinischen Wissenschaften (2020) Covid-19-Pandemie: Triage von intensivmedizinischen Behandlungen bei Ressourcenknappheit. https://www.samw.ch/de/Ethik/Themen-A-bis-Z/Intensivmedizin.html. Accessed 30 June 2020 (Aktualisierte Version vom 24.03.2020)

[19] Truog RD, Mitchell C, Daley GQ (2020). The toughest triage—Allocating ventilators in a pandemic. N Engl J Med.

[20] Vincent JL, Moreno R, Takala J (1996). The SOFA (Sepsis-related Organ Failure Assessment) score to describe organ dysfunction/failure. On behalf of the Working Group on Sepsis-Related Problems of the European Society of Intensive Care Medicine. Intensive Care Med.

[21] White DB (2020) A model hospital policy for allocation of scarce critical care resources. University of Pittsburgh School of Medicine. https://ccm.pitt.edu/?q=content/model-hospital-policy-allocating-scarce-critical-care-resources-available-online-now. Accessed 30 June 2020

[22] White DB, Lo B (2020). A framework for rationing ventilators and critical care beds during the COVID-19 pandemic. JAMA.

