# Supplementary material for: Decisions on the allocation of intensive care resources in the context of the COVID-19 pandemic: Clinical and ethical recommendations of DIVI, DGINA, DGAI, DGIIN, DGNI, DGP, DGP and AEM
Source: Med Klin Intensivmed Notfmed. 2020 Jul 29;115(Suppl 3):115–22. doi: 10.1007/s00063-020-00709-9 (PMC7387419; doi:10.1007/s00063-020-00709-9)
Supplement: Supplementary file 1 — Fig. 1 Documentation support for prioritisation in case of resource scarcity [file 63_2020_709_MOESM1_ESM.pdf]

Fig. 1 Documentation support for prioritisation in case of resource scarcity

Patient label

Date/Time

Team members (Name/Function)

Clinical ethics involved? yes ☐ /no ☐

Need for intensive care therapy

Evaluation of clinical prospect of success of intensive care therapy

Current illness	General health status (prior to current illness)
Prognosis score 1)	General health score 2)
SCORE / PTs	SCORE / PTs

according **to relevant clinical measure**; 1) e.g. SOFA, APACHE II or CRB-65; 2) e.g. Clinical Frailty Scale CFS or ECOG

Comorbidities

Reducing the chance of surving intensive care due to severity or combination:

- ☐ severe organ dysfunction
  - ☐ heart ☐ lung ☐ liver ☐ kidney
- ☐ advanced neurological disease
- ☐ advanced oncological disease
- ☐ severe and irreversible immune deficiency
- ☐ multimorbidity

Comments:

Patient‘s wishes

Conversation with patient (current wishes) ☐
Advance directive available? yes ☐ /no ☐
Power of attorney available? yes ☐ /no ☐

Conversation with
☐ *legal representative:*

☐ *Relatives (if no legal representative appointed)*

☐ No consent to intensive care

☐ Consent to intensive care

☐ Patient‘s wishes cannot be determined

TRIAGE Result

Intensive Care		Non-Intensive Care	
☐ ICU	☐ Intermediate Care	☐ General Ward	☐ Palliative Care

RE-EVALUATION on (Date/Time):

Assessment of clinical course of disease:

Continuation or withdrawal of therapy because:
