# Supplementary material for: Decisions on the allocation of intensive care resources in the context of the COVID-19 pandemic: Clinical and ethical recommendations of DIVI, DGINA, DGAI, DGIIN, DGNI, DGP, DGP and AEM
Source: Med Klin Intensivmed Notfmed. 2020 Jul 29;115(Suppl 3):115–22. doi: 10.1007/s00063-020-00709-9 (PMC7387419; doi:10.1007/s00063-020-00709-9)

Fig. 2 Decision-making in case of insufficient intensive care resources

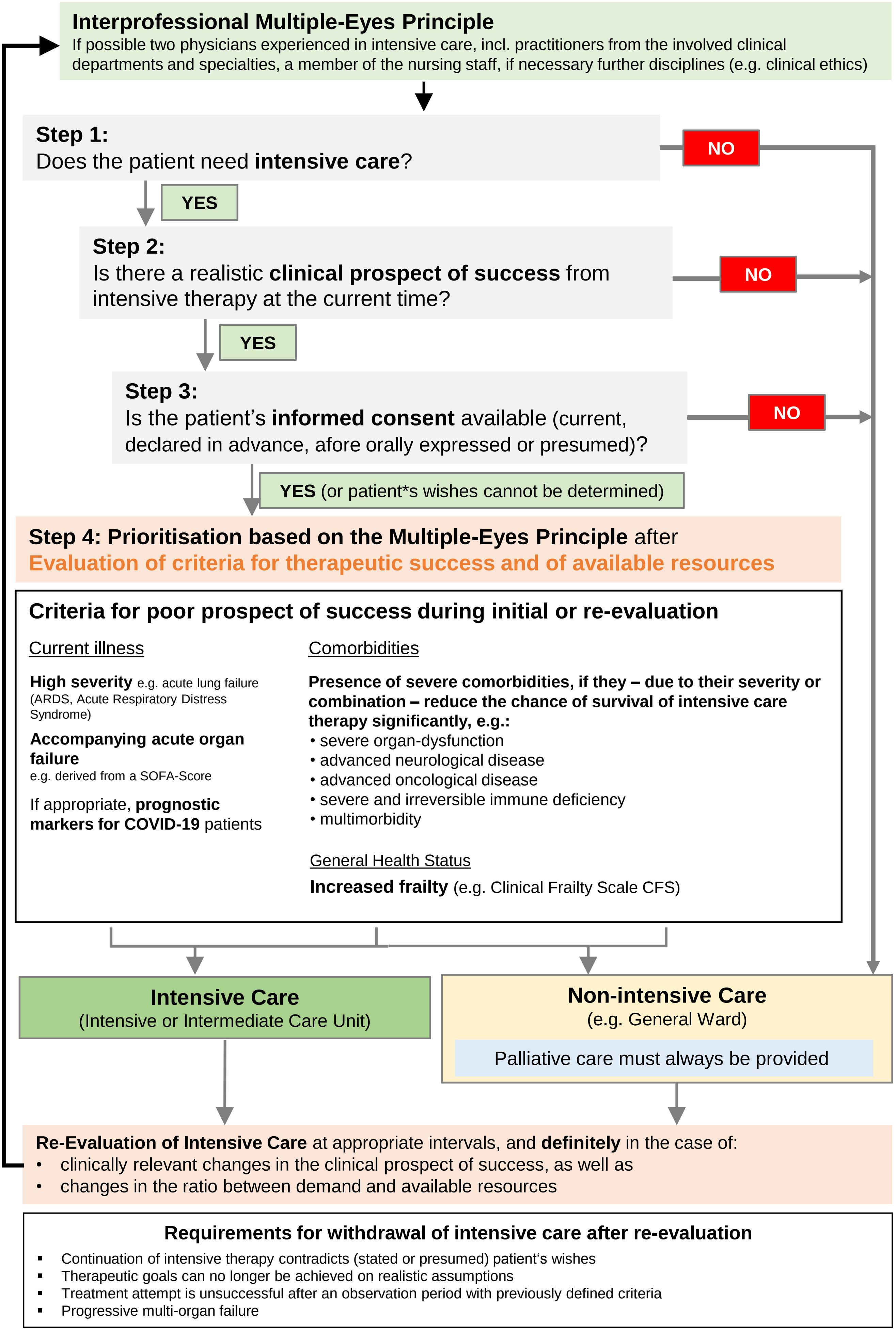

Supplement: Supplementary file 2 — Fig. 2 Flowchart—decision-making in the case of insufficient intensive care resources [file 63_2020_709_MOESM2_ESM.pdf]
